# Supplementary material for: Flashzoi: an enhanced Borzoi for accelerated genomic analysis
Source: Bioinformatics. 2025 Sep 4;41(9):btaf467. doi: 10.1093/bioinformatics/btaf467 (PMC12457734; doi:10.1093/bioinformatics/btaf467)
Supplement: btaf467_Supplementary_Data [file btaf467_supplementary_data.pdf]

**Layer Name**	**Borzoi (1 replicate)**	**Flashzoi (1 replicate)**
DNA convolution	31,232	31,232
Convolutional tower	1,558,112	1,558,112
	2,239,392	2,239,392
	3,299,648	3,299,648
	4,733,728	4,733,728
	6,761,792	6,761,792
	9,834,496	9,834,496
Unet skip connection 1	1,967,616	1,967,616
	2,560	2,560
Unet skip connection 2	2,360,832	2,360,832
	3,072	3,072
Transformer tower	**15,758,336**	**16,530,432**
	**15,758,336**	**16,530,432**
	**15,758,336**	**16,530,432**
	**15,758,336**	**16,530,432**
	**15,758,336**	**16,530,432**
	**15,758,336**	**16,530,432**
	**15,758,336**	**16,530,432**
	**15,758,336**	**16,530,432**
Upsampling layer 1	2,363,904	2,363,904
	2,365,440	2,365,440
Upsampling layer 2	2,363,904	2,363,904
	2,365,440	2,365,440
Joined pointwise convolutions	2,954,112	2,954,112
Human head	14,620,731	14,620,731
Mouse head	5,009,968	5,009,968
**Total Trainable Parameters**	**190,902,667**	**197,079,435**

Supplementary Table S1. Parameter count of the Borzoi and Flashzoi models.
